# Cyclic Phosphatidic Acid Stimulates cAMP Production and Inhibits Growth in Human Colon Cancer Cells

**DOI:** 10.1371/journal.pone.0081139

**Published:** 2013-11-25

**Authors:** Tamotsu Tsukahara, Yoshikazu Matsuda, Hisao Haniu

**Affiliations:** 1 Department of Hematology and Immunology, Kanazawa Medical University, 1-1, Daigaku, Uchinada, Ishikawa, Japan; 2 Clinical Pharmacology Educational Center, Nihon Pharmaceutical University, Ina-machi, Saitama, Japan; 3 Department of Orthopaedic Surgery, Shinshu University School of Medicine, 3-1-1 Asahi, Matsumoto, Nagano, Japan; Rush University Medical Center, United States of America

## Abstract

Colon cancer is a malignancy that develops in colon and rectal tissues. The prognosis for metastatic colon cancer remains poor, and novel therapeutic options are required to reduce colon cancer mortality. Recently, intracellular cAMP levels have been suggested to influence the behavior of cancer cells. Intriguingly, cyclic phosphatidic acid (cPA) and its structural analogs inhibit growth in many cancer cell lines, and our previous work has suggested that cPA increases cAMP production. Phosphodiesterase (PDE) type 3 isoforms PDE3A and PDE3B are expressed mainly in cardiovascular tissue and adipose tissue, respectively. Moreover, increase in intracellular cAMP levels has been associated with the inhibition of growth in colon cancer cells. These findings suggest that cPA could be used in colon cancer therapy. In this study, we found that cPA inhibited the growth of HT-29 cells, which express high levels of PDE3B, but not the growth of DLD-1 cells, which express low levels of PDE3B. Furthermore, cPA inhibited the phosphorylation of Akt in HT-29 cells in a dose-dependent fashion. Our results suggest that PDE3B expression and intracellular cAMP levels are correlated with the proliferation of colon cancer cells. These findings demonstrate for the first time that cPA may serve as a useful a molecule in targeted therapy for colon cancer.

## Introduction

Cyclic nucleotide phosphodiesterase (PDE) is an enzyme that breaks phosphodiester bonds [1]. In humans, PDEs are coded by 21 PDE genes [1], which are divided into 11 families based on structural similarity such as protein sequence homology. The PDEs comprise a group of enzymes that cleave the phosphodiester bond in the second messenger cAMP, which plays a central role in cellular responses to diverse extracellular stimuli [2]. Intracellular cAMP levels are regulated normally by the balance between the activities of two types of enzymes, the cAMP-generating enzymes (adenylate cyclases) and the cAMP-degrading enzymes (PDEs), mainly in response to hormones and neurotransmitters [3] [4]. PDE3B was identified in humans and its transcripts are found predominantly in adipose tissue [5], and PDE3B has been reported to be phosphorylated and activated in response to insulin and hormones that increase cAMP levels [6].

Bioactive lipids such as cyclic phosphatidic acid (cPA) [7] [8] have been suggested to increase cellular cAMP levels and lead to RhoA inactivation in hepatoma cells [9]. Previously, we showed that cPA reduced intracellular triglyceride levels and inhibited PDE3B expression [8]. Moreover, intracellular cAMP levels in 3T3-L1 cells were found to increase after exposure to cPA. These results suggest the cPA-PDE3B-cAMP pathway is a specific molecular target. Phospholipase D2 (PLD2) generates cPA from lysophosphatidylcholine (LPC), and low-dose insulin treatment of cells stimulates PLD2 activity and increases intracellular cPA levels [7].

Recently, PDE3 has been suggested to play a key role in cancer cell invasion and cell motility [10]. PDE3 inhibitors such as cilostazol inhibited the growth of small-cell lung carcinoma cells [11], identifying PDE3 as a target for anti-proliferative cancer therapy. Reduction in PDE3B activity is accompanied by increases in intracellular levels of cAMP, which activates cAMP-dependent protein kinase A (PKA) [1]. In normal human cells, cAMP promotes proliferation and differentiation, but in cancer cells, cAMP affects proliferation distinctly and suppresses basal proliferation to levels considerably than in normal human cells [12]. Moreover, high intracellular levels of cAMP can effectively reduce in vitro cancer cell growth [1]. 

Akt (Protein kinase B) has been shown recently to attenuate cAMP signaling by activating PDE3B [13]. Since its discovery as a proto-oncogene, the serine/threonine kinase Akt has become a major focus of attention because Akt regulates diverse cellular processes critically, including cancer progression [14]. In cancer, Akt activity is frequently elevated because of multiple mechanisms, including the loss of function of the PTEN tumor suppressor gene [15]. When activated, Akt can phosphorylate multiple downstream molecules involved in regulating cell proliferation and suppressing apoptosis [16]. Akt signaling is linked to tumor formation, and Akt inhibitors have been developed to control cancer growth [14]. However, for colon cancer, therapeutic options are currently limited because these treatments produce adverse cardiovascular and thrombotic effects [17]. Thus, other signaling pathways must be considered that can be used to develop new therapeutic strategies to target colon cancer. Intriguingly, cPA has been reported to produce anti-mitogenic effects and prevent cancer cell invasion in vitro and metastasis in vivo [18] [19].

We investigated the expression of PDE3 isoforms PDE3A and 3B in human colon cancer cell lines HT-29 and DLD-1. Real-time polymerase chain reaction (RT-PCR) and western blotting revealed that PDE3D was the only PDE3 isoform expressed in both cell lines. PDE3B mRNA and protein were expressed at high levels in HT-29 cells, and we observed that PDE3B expression levels correlated with colon cancer cell proliferation. We found that cPA treatment elevated intracellular cAMP and suppressed the proliferation of HT-29 cells. Furthermore, cPA inhibited Akt phosphorylation in HT-29 cells in a dose-dependent fashion. Together, these results suggest that the cPA-PDE3B-cAMP pathway plays a critical in the progression of colon cancer.

## Materials and Methods

### Reagents

cPA (18:1) was purchased from Avanti Polar Lipids Inc. (Alabaster, AL). Dexamethasone was purchased from Sigma-Aldrich (St. Louis, MO). Cilostazol, anti-PDE3B antibody (sc-20793), and anti-β-actin antibody (sc-47778) were purchased from Santa Cruz Biotechnology (Santa Cruz, CA). Anti-Akt (#9272) and anti-pAkt S473 (9271S) were purchased from Cell Signaling Technology (Danvers, MA).

### Cell culture

Human colon cancer cell lines HT-29, LOVO, and Caco-2 were obtained from American Type Culture Collection (Manassas, VS). DLD-1 human adenocarcinoma cells were obtained from the Health Science Research Resources Bank (Osaka, Japan). Cells were grown in Dulbecco’s Modified Eagle Medium (DMEM; Nacalai Tesque, Kyoto, Japan) or RPMI-1640 medium (Nacalai Tesque) containing 10% (v/v) fetal bovine serum (FBS), 100 U/mL penicillin, 10 μg/mL streptomycin, and 2.5 μg/mL plasmocin^TM^ (Nacalai Tesque) at 37°C in a humidified incubator with 5% CO_2_. 

### Western blot analysis

 Cells were seeded in 6-well plates (Iwaki, Tokyo, Japan) at a density of 1 × 10^5^ cells/well. After various treatments (as indicated), cells were lysed on ice for 30 min in a cell-lysis buffer (20 mM Tris-HCl [pH 7.4], 10% [v/v] glycerol, 100 mM NaCl, 1% [v/v] Triton X-100, 1/100 protease inhibitor cocktail [Sigma], 1 mM dithiothreitol) and centrifuged at 16,000 × *g* for 20 min at 4°C. The supernatants were saved as cell lysates and assayed for protein content using the Bradford method (Bio-Rad Protein Assay kit; Bio-Rad Laboratories, Hercules, CA). Cell lysates were then separated on 5%–20% sodium dodecyl sulfate (SDS)-polyacrylamide gels (e-PAGEL; ATTO, Tokyo, Japan) and electrotransferred to Immobilon-P membranes (Millipore, Billerica, MA). The membranes were blocked for 1 h with Block Ace (DS Parma Biomedical Co. Ltd., Osaka, Japan) and incubated with primary antibodies diluted in TBS-T with 5% Block Ace for 12 h at 4°C. Protein bands were visualized using EzWestLumi plus (ATTO).

### Quantitative real-time PCR analysis

Total cellular RNA was prepared using NucleoSpin RNA II (Takara, Shiga, Japan), and 0.5 μg of total RNA was used for synthesizing cDNA with the ReverTra Ace qPCR RT Kit (Toyobo, Osaka, Japan) according to the manufacturer’s recommendations. We measured mRNA levels using an ECO Real-Time PCR system (Illumina Inc., San Diego, CA) and SYBR Green Realtime PCR Master Mix-Plus (Toyobo) with the following primer pairs being used in the reactions: PDE3A, 5′-AAAGACAAGCTTGCTTGCTATTCCAAA-3′ (F) and 5′-GTGGAAGAAACTCGTCTCAACA-3′ (R); PDE3B, 5′-CCAGGTGTGCATCAAATTAGCA-3′ (F) and 5′-CAATGCCTTCTGTCCATCTCAA-3′ (R); 18S rRNA, 5′- CAGCCACCCGAGATTGAGCA-3′ (F) and 5′-TAGTAGCGACGGGCGGTGTG-3′ (R). All PCR reactions were performed in a 10 μL volume, using 48-well PCR plates (Illumina). The cycling conditions were 95°C for 10 min (enzyme activation) followed by 40 cycles of 95°C for 15 s, 55°C for 15 s, and 72°C for 30 s. After amplification, the samples were heated slowly from 55°C to 95°C and the fluorescence was measured continuously to obtain a melting curve. Relative mRNA levels were calculated using the formula 2^-ΔΔCq^, where ΔCq is the difference between the threshold cycle of a given target cDNA and that of an endogenous reference cDNA. Derivation of the formulas and the validation tests have been described in Applied Biosystems User Bulletin No. 2.

### Measurement of cell proliferation

Cells were seeded in 96-well culture plates (5 × 10^3^ cells/well) and incubated for 24 h. Cell proliferation was measured using the Cell Counting Kit-8 (Dojindo, Kumamoto, Japan): 10 μL of Cell Counting Kit-8 solution was added to the medium and incubated for 2 h in an incubator with 5% CO_2_; the amount of orange formazan dye produced was calculated by measuring the absorbance at 450 nm in a microplate reader (Awareness Technology, Inc., Palm City, FL).

### Cyclic nucleotide phosphodiesterase assay

The activity of recombinant PDE3B tagged with GST (BPS Bioscience Inc., San Diego, CA) was assayed using a cyclic nucleotide PDE assay kit (Enzo Life Science, Farmindale, NY) in 96-well plates and measuring absorbance at 620 nm with a spectrophotometer; assays were performed according to the manufacturer’s protocol.

### Effect of cPA on intracellular cAMP level

Cells were cultured in serum-free medium containing cPA for 60 min. Cellular cAMP levels were measured using ELISA (cAMP Biotrak Enzyme immunoassay system, Amersham Biosciences) in 96-well plates and by determining the absorbance at 450 nm with a spectrophotometer by following the manufacturer’s instructions.

### RNA interference

We suppressed PDE3B and Akt expression in HT-29 cells by transfecting the cells with small interfering RNAs (siRNAs) targeting PDE3B and Akt (Santa Cruz Biotechnology); Lipofectamine RNAiMAX (Invitrogen) was used for transfections. Cells were plated in 6-well plates (Iwaki) at a density of 5 × 10^4^ cells/well in DMEM containing 10% FBS and then transfected with 100 pmol/mL of mRNA-specific siRNAs or scrambled (control) siRNAs. Reduction of PDE3B and Akt levels was confirmed by western blotting.

### Statistical analysis

Student’s *t*-test was used for statistical comparisons. Differences were considered significant at the *p* < 0.05 level.

## Results

**Figure 1:**
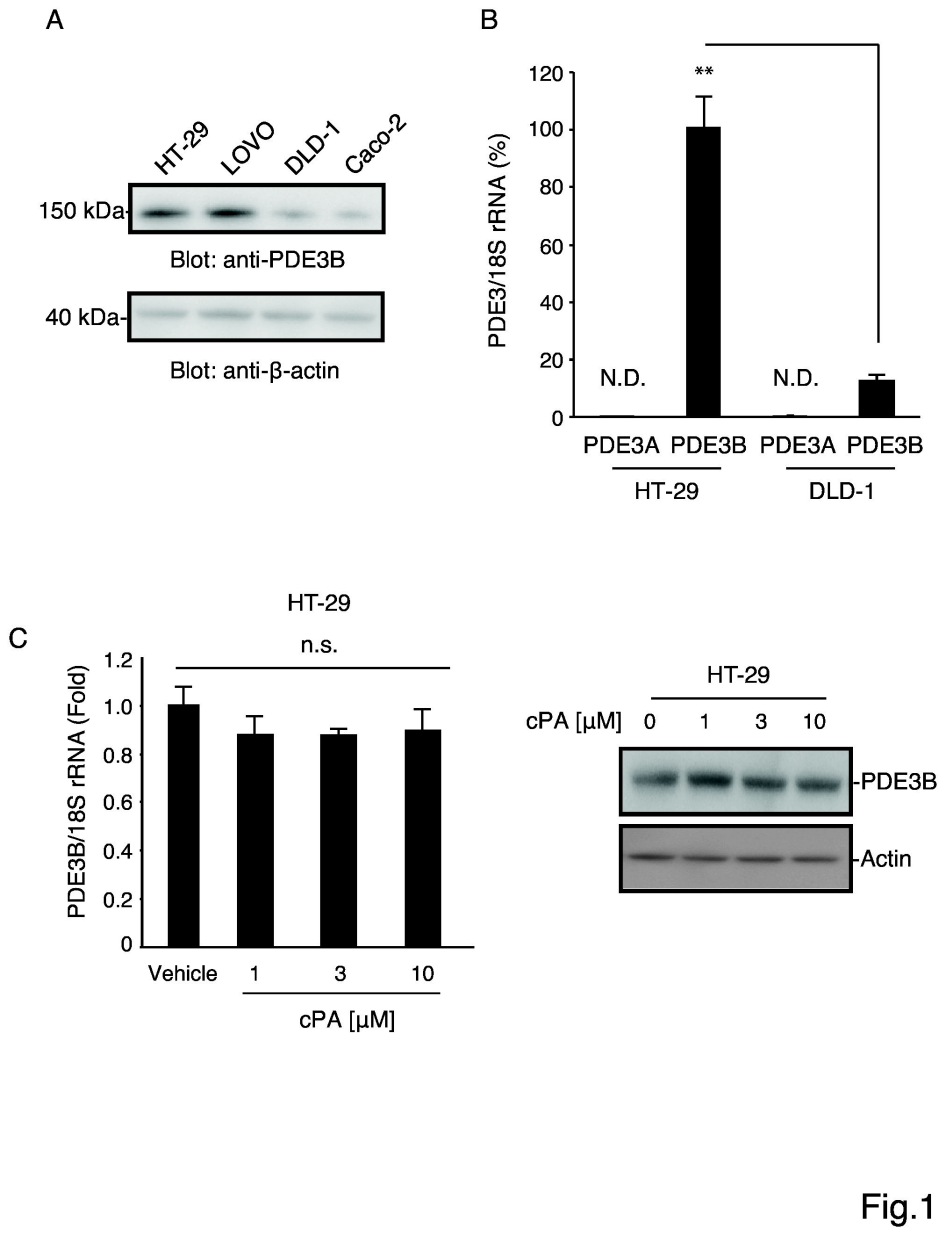
(A) Representative western blots showing PDE3B expression. Four human colon cancer cell lines (HT-29, LOVO, DLD-1, and Caco-2 cells) were cultured in DMEM supplemented with 10% FBS; protein levels were analyzed using SDS-PAGE and western blotting with an anti-PDE3B antibody, and protein bands were visualized using an enhanced chemiluminescence reagent. Each lane was loaded with 20 μg of whole-cell lysate. β-actin was stained as a loading control. (B) Real-time PCR measurement of the expression of PDE3A and 3B mRNAs and 18S rRNA (internal control) in HT-29 and DLD-1 cells (mean ± SEM, n = 3, **p < 0.01, Student’s *t* test). (C) Real-time PCR analysis (left) and western blotting (right) for comparing PDE3B expression in HT-29 cells after treatment with cPA (1, 3, and 10 μM) for 60 min (mean ± SEM, n = 3, **p < 0.01, Student’s *t* test).

The PDE3 isoforms PDE3A and PDE3B are known to be expressed in humans [1]. To evaluate the function of PDE3 in human colon cancer cells, we used 4 colon cancer cell lines, HT-29, LOVO, DLD-1, and Caco-2 (Figure 1A), and examined the expression of PDE3B protein in these cell lines. PDE3B was expressed at high levels in HT-29 and LOVO cells.

**Figure 2:**
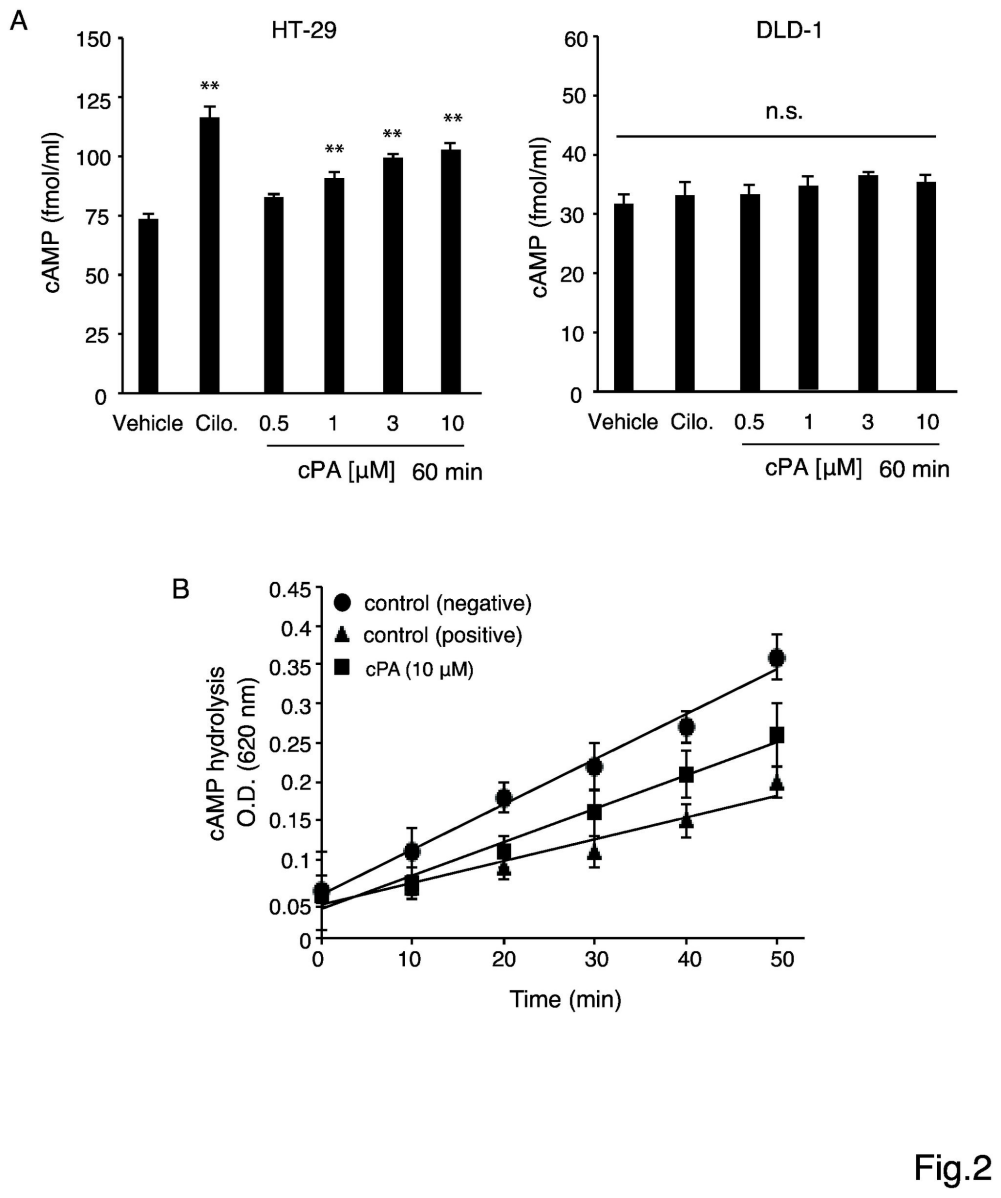
(A) Intracellular cAMP levels in HT-29 and DLD-1 cells treated with 0.5, 1, 3, or 10 μM cPA for 60 min. Intracellular cAMP levels in culture lysates were measured using the cAMP Biotrak Enzyme immunoassay system. Cilostazol (5 μM; Cilo) was used as a positive control. Data are expressed as means ± SEM (n = 4), **p < 0.01. (B) Time course of cPA-dependent inhibition of cAMP hydrolysis by PDE3B. PDE3B was incubated with cAMP and 5′-nucleotidase with or without the cPA (10 μM) for 10–50 min; the PDE inhibitor IBMX (50 μM) was used as a positive control.

For further studies, we chose one cell line expressing high levels of PDE3B, HT-29, and one cell line expressing low levels of PDE3B, DLD-1. Agreeing with the protein expression results, PDE3B mRNA was also present at higher levels in HT-29 cells than in DLD-1 cells (Figure 1B). By contrast, PDE3A mRNA was not detected in either cell lines. These results suggest that PDE3B is the major PDE isoform in HT-29 and DLD-1 cells. We tested the effect of cPA on the expression of PDE3B mRNA and protein in HT-29 cells (Figure 1C), and found that cPA did not influence PDE3B gene expression, suggesting that cPA does not produce its effects by diminishing PDE3B expression in HT-29 cells. Next, we examined the effect of cPA on intracellular cAMP levels in HT-29 and DLD-1 cells. Although cAMP can either promote or suppress proliferation in many cell types, in most cases cAMP appears to be anti-proliferative. Treatment with cPA increased cAMP levels substantially in HT-29 cells but not in DLD-1 cells (Figure 2A). The above results indicated that the level of PDE3B expression was correlated with the proliferation potential of colon cancer cells. Interestingly, time-course experiments showed that cPA treatment inhibited cAMP hydrolysis by PDE (Figure 2B), suggesting that blocking PDE3 activity with cPA enhances intracellular cAMP accumulation. However, cAMP may exert its anti-proliferative effect through distinct mechanisms in various cell lines.

**Figure 3:**
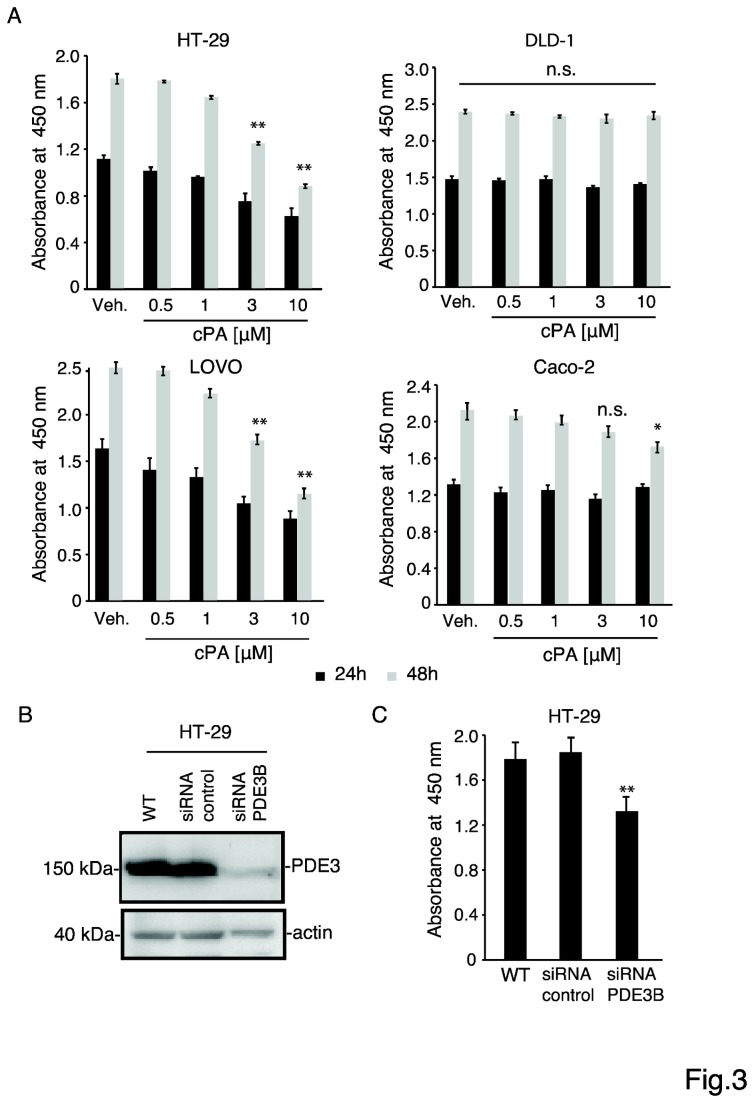
(A) Dose-dependent inhibition of cell growth by cPA was evaluated using the Cell Counting Kit-8. Cells (1 × 10^5^ cells/well) were seeded in 6-well plates and incubated for 24 h or 48 h at 37°C with 5% CO_2_, after which 10 μL of the Cell Counting Kit-8 solution was added to the medium. After incubating for 2 h more, the amount of orange formazan dye generated was determined by measuring the absorbance at 450 nm using a microplate reader. Data are expressed as means ± SEM (n = 4), **p < 0.01. (B) Knocking down PDE3B expression in HT-29 cells. Total protein was extracted from untransfected cells and from cells transfected with control siRNA or PDE3B-specific siRNA and analyzed by western blotting with an anti-PDE3B antibody; β-actin was stained as a protein-loading control. (C) Inhibition of cell growth following PDE3B knockdown was measured using the Cell Counting Kit-8. Cells (1 × 10^5^ cells/well) were seeded in 6-well plates and incubated for 24 h at 37°C with 5% CO_2_, after which 10 μL of the Cell Counting Kit-8 solution was added to the medium. After incubating for 2 h more, the amount of orange formazan dye generated was determined by obtaining the absorbance at 450 nm using a microplate reader. Data are expressed as means ± SEM (n = 4), **p < 0.01.

The effect of cPA on cell proliferation was determined using a colorimetric assay. We found that cPA inhibited the proliferation of HT-29 and LOVO cells but not of DLD-1 and Caco-2 cells (Figure 3A), which have lower endogenous levels of PDE3B than HT-29 and LOVO cells [8]. To test whether PDE3B affects the proliferation of HT-29 cells, PDE3B expression was knocked down using siRNAs. The PDE3B-targeting siRNA knocked down PDE3B effectively, as shown by western blotting with an anti-PDE3B antibody (Figure 3B). Notably, 24 h after transfecting with the PDE3B-siRNA, the proliferation of HT-29 cells was decreased substantially. These results indicate that knocking down PDE3B inhibits the growth of HT-29 cells.

**Figure 4:**
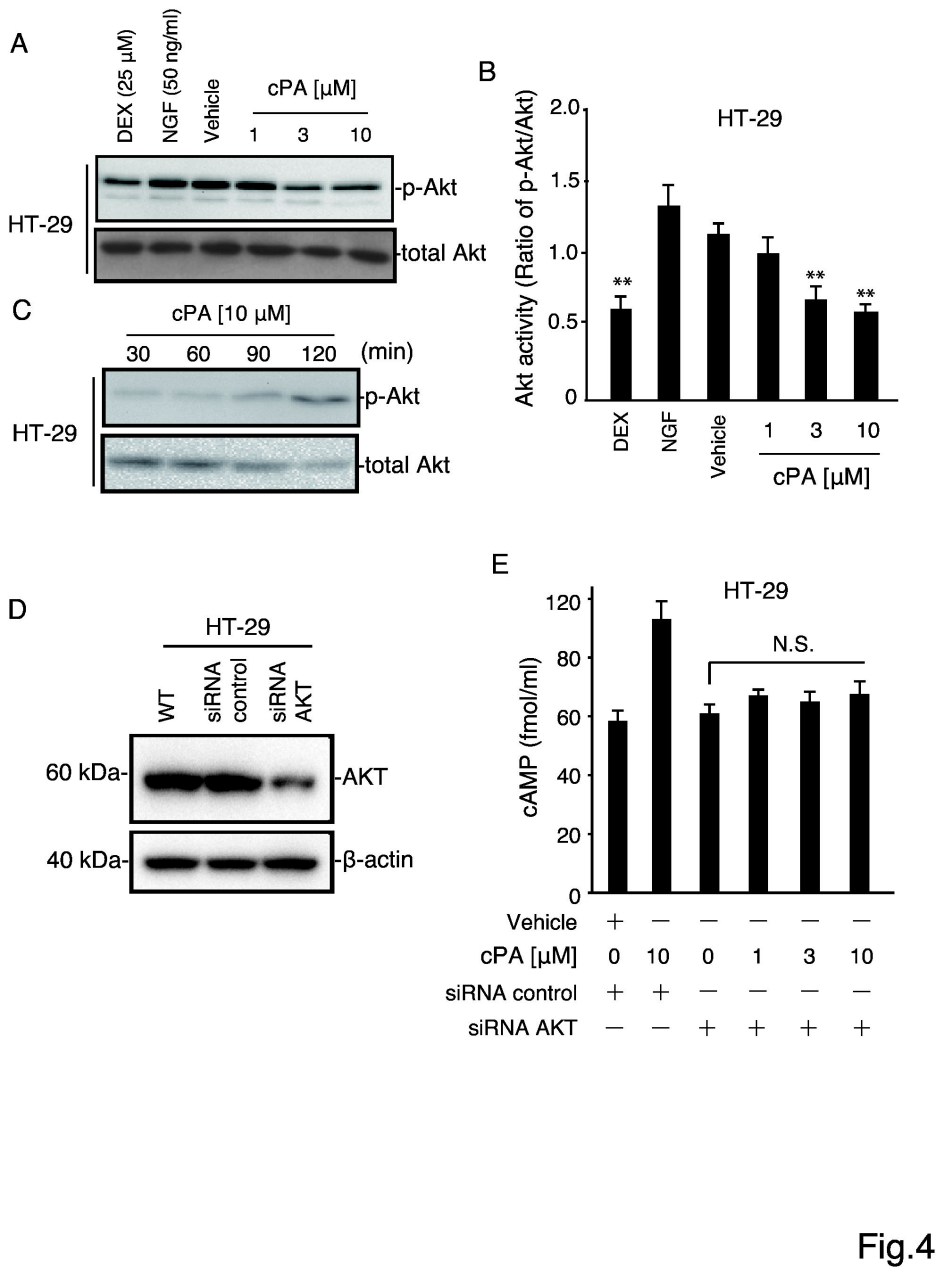
Inhibition of Akt phosphorylation in HT-29 cells by cPA. (A) Akt phosphorylation in HT-29 cells treated with cPA (1, 3, and 10 μM), NGF (50 ng/mL, positive control), or dexamethasone (Akt inhibitor, 25 μM). Phosphorylated Akt (p-Akt) and total Akt were detected by immunoblotting. (B) The intensities of the Akt bands were quantified, and the ratio of phosphorylated to total Akt was calculated. Data are expressed as means ± SEM (n = 3), **p < 0.01. (C) HT-29 cells were treated with cPA (10 μM) and lysates were collected at 30, 60, 90, and 120 min. Akt inhibition was measured as a loss of Ser473 phosphorylation. (D) Knocking down Akt expression in HT-29 cells. Total protein was extracted from untransfected cells and from cells transfected with control siRNA or Akt-specific siRNA and analyzed by western blotting with an anti-Akt antibody; β-actin was stained as a protein-loading control. (E) Intracellular cAMP levels in HT-29 treated with 1, 3, or 10 μM cPA for 60 min after Akt knockdown; cAMP levels were determined in cell lysates using the cAMP Biotrak Enzyme immunoassay system. Data are expressed as means ± SEM (n = 3), **p < 0.01.

Our results suggest that PDE3B levels correlates with cell proliferation rates and that the effect of PDE3B depends on the cell type. Next, we determined whether cPA inhibits Akt phosphorylation in HT-29 cells, because many effects of cAMP are mediated through the activation of Akt [20] [21]. Akt is associated with tumor-cell survival, proliferation, and invasiveness [22] [23]. To ascertain whether cPA contributes to the effects of Akt in HT-29 cells, we tested how cPA affects Akt activation. Inhibition of Akt signaling has been associated with the biological actions of chemo-preventive compounds such as epigallocatechin gallate, which markedly suppresses pAkt levels in intestinal tumors without altering total Akt levels substantially [24]. Akt activation involves the phosphorylation of two residues, Thr308 in the activation loop and Ser473 in the C-terminal hydrophobic motif; phosphorylation of Ser473 has been examined widely in tumor samples as an indicator of Akt activity [25]. First, we examined baseline Akt Ser473 phosphorylation in HT-29 cells and found this site in Akt to be constitutively phosphorylated (Figure 4A). Moreover, we determined whether cPA inhibited Akt phosphorylation in HT-29 cells. Our results indicated that cPA blocked the constitutive phosphorylation of Akt in a dose-dependent fashion (Figure 4, A and B), and that this cPA-mediated inhibition of Akt phosphorylation lasted up to 120 min (Figure 4C). Although cPA is a stable lipid and 75% of the molecule can remain intact in culture medium for 24 h [19], cPA can be converted to LPA by the opening of the ring structure of cPA. This raises the possibility that hydrolytic cleavage of the cyclic phosphate ring of cPA by phospholipid phosphatases in HT-29 cells leads to the formation LPA, which can activate Akt [26].

Lastly, we measured cAMP levels in HT-29 cells in the presence and absence of cPA after knocking down Akt; western blotting with an anti-Akt antibody confirmed that the Akt-targeting siRNA knocked down Akt expression effectively in HT-29 cells (Figure 4D). Notably, cPA treatment of the HT-29 cells with diminished Akt levels failed to increase cAMP levels significantly (Figure 4E). 

## Discussion

Lysophospholipid has long been recognized as a membrane phospholipid metabolite. Recently, however, the lysophospholipid has emerged as a candidate molecule for diagnostic and pharmacological purposes, and LPA has been reported to be a potent inducer of cancer progression at multiple levels. Although cPA is similar chemically to LPA, the functions of cPA are distinct from or even opposite to those of LPA. For example, LPA stimulates but cPA inhibits cell proliferation and cancer-cell invasion [19] [27] [9]. Moreover, cPA can suppress cancer invasion and raise intracellular cAMP levels [27]. PDE3 enzymes constitute one of the most extensively studied families of cAMP-hydrolyzing PDEs, because PDE3 enzymes play several roles in physiological and pathophysiological processes in cancer [28]. In the context of colon cancer, the PDE3-specific inhibitor cilostazol, which has been used previously to treat patients with thrombosis, was used to assess the effects of PDE3B inhibition on cellular growth. Proliferation of cells is inhibited by cAMP through diverse mechanisms that can induce cell-cycle arrest at G1 and apoptosis [29]. However, the mechanism through which PDE3B is involved in intracellular cAMP production in response to cPA has remained unclear. We conducted this study on colon cancer cells using cPA, which had been shown previously to increase intracellular cAMP levels directly [8], a function of cPA that is also suggested by the anti-invasive properties of cPA [9]. We found that cPA inhibited the growth of HT-29 and LOVO cells, which express high levels of PDE3B, but not the growth of DLD-1 and Caco-2 cells, which express low levels of PDE3B. Supporting these findings, cPA inhibited PDE3B activity in cells expressing high levels of PDE3B, and siRNA-mediated suppression of PDE3B expression inhibited cell growth. These results suggest that PDE3B regulates intracellular cAMP levels in colon cancer cells and is involved in cancer cell growth. In this study, we found that cPA inhibited the phosphorylation of Akt in HT-29 cells in a dose-dependent fashion. Akt regulates multiple cellular functions including cell survival and proliferation and various aspects of intermediary metabolism. In the neoplastic colonic epithelium, Akt was found to be not only expressed at higher levels but also hyperactivated [30], and studies on chemical models have confirmed that Akt is upregulated in the early stages of intestinal tumorigenesis. PDE3B expression and intracellular cAMP levels are correlated with the proliferation potential of colon cancer cells. We corroborated our findings using siRNA analysis and demonstrated a decrease in pSer473-Akt with good correlation between degree of cAMP production and downregulation of Akt phosphorylation. These results suggest that Akt related signaling pathway is tightly linked to the cPA-PDE3B-cAMP pathway and thus indicate for the first time that cPA may serve as a useful molecule in targeted therapy for colon cancer. 

## Conclusions

Our findings indicate that cAMP plays a critical role in the inhibition of colon cancer cell growth by cPA. Based on our results, we suggest that elucidating the molecular mechanisms by which cPA induces cAMP production by inhibiting PDE3B in colon cancer cells can provide valuable insights that help explain how cancer cell growth is regulated. Understanding how cancer cell growth is regulated would, in turn, help unravel the molecular mechanisms of cancer-cell invasion and metastasis and facilitate the analysis of the signal transduction pathways that lead to cell proliferation. Although further research is required for examining the crosstalk between the signaling molecules we have described here, our results collectively support the potential use of cPA as a therapeutic compound for treatment of colon cancer.
